# The α1 Antagonist Doxazosin Alters the Behavioral Effects of Cocaine in Rats

**DOI:** 10.3390/brainsci2040619

**Published:** 2012-11-13

**Authors:** Colin N. Haile, Yanli Hao, Patrick O’Malley, Thomas F. Newton, Therese A. Kosten

**Affiliations:** Michael E. DeBakey VA Medical Center, Menninger Department of Psychiatry & Behavioral Sciences, Baylor College of Medicine, Houston, TX 77030, USA; Email: chaile@bcm.edu (C.N.H.); haoyanli26@yahoo.com (Y.H.); pwomalle@bcm.edu (P.O.); tnewton@bcm.edu (T.F.N.)

**Keywords:** cocaine dependence, psychostimulants, norepinephrine, dopamine, locomotor sensitization

## Abstract

Medications that target norepinephrine (NE) neurotransmission alter the behavioral effects of cocaine and may be beneficial for stimulant-use disorders. We showed previously that the short-acting, α1-adrenergic antagonist, prazosin, blocked drug-induced reinstatement of cocaine-seeking in rats and doxazosin (DOX), a longer-acting α1 antagonist blocked cocaine’s subjective effects in cocaine-dependent volunteers. To further characterize DOX as a possible pharmacotherapy for cocaine dependence, we assessed its impact on the development and expression of cocaine-induced locomotor sensitization in rats. Rats (*n* = 6–8) were administered saline, cocaine (COC, 10 mg/kg) or DOX (0.3 or 1.0 mg/kg) alone or in combination for 5 consecutive days (development). Following 10-days of drug withdrawal, all rats were administered COC and locomotor activity was again assessed (expression). COC increased locomotor activity across days indicative of sensitization. The high dose (1.0 mg/kg), but not the low dose (0.3 mg/kg) of DOX significantly decreased the development and expression of COC sensitization. DOX alone did not differ from saline. These results are consistent with studies showing that α1 receptors are essential for the development and expression of cocaine’s behavioral effects. Results also suggest that blockade of both the development and expression of locomotor sensitization may be important characteristics of possible pharmacotherapies for cocaine dependence in humans.

## 1. Introduction

Cocaine addiction is a chronic relapsing brain disease for which there are no FDA-approved treatments. The prevailing consensus is that cocaine’s powerful reinforcing effects in humans (e.g., euphoria, heightened alertness, increased energy levels) are mediated by its ability to increase central monoamines (dopamine, DA, norepinephrine, NE, and serotonin) via transporter blockade within the mesocorticolimbic system [1,2]. This system consists of DAergic neurons in the ventral tegmentum (VTA) that project to the nucleus accumbens (NAc) and prefrontal cortex (PFC) among other areas. Increases in synaptic DA levels within the NAc are critical to cocaine’s powerful reinforcing effects [3,4,5]. Neuroplastic changes secondary to chronic cocaine exposure result in augmented NAc DA and behavior termed sensitization that is hypothesized to relate to various aspects of dependency in humans [6,7,8]. Unfortunately, decades of preclinical and clinical drug development research focused on DA has not lead to a single medication with proven efficacy prompting the consideration of other possible therapeutic targets [9]. 

A much-neglected facet of cocaine’s action is its effects on brain NE and its relationship with, and ability to modulate, DA neurotransmission [10]. For example, NEergic fibers from the A2 region of the nucleus tractus solitarius and the primary NE cell body region, locus coeruleus (LC), innervate the shell region of the NAc where DA plays a critical role in mediating the behavioral effects of cocaine [4,11]. DA and NE are co-released in cortical brain regions where NE transporters can sequester DA [12,13]. LC projections also innervate the VTA and PFC. Accordingly, ablating the LC attenuates stimulant-induced locomotor activation [14]. Under certain circumstances descending excitatory PFC projections responsible for DA release in the NAc are influenced by NE [15]. Moreover, depletion of NE in the PFC eliminates stimulant-induced DA release in the NAc and cocaine-induced locomotor activation [16]. Consistent with these findings, studies in animals and humans show disulfiram, a drug that eliminates NE by inhibiting the enzyme dopamine β-hydroxylase responsible for its synthesis, alters cocaine’s behavioral effects in animals and modestly decreases cocaine use in humans [17,18]. 

Accumulating evidence supports the notion that the behavioral effects of psychostimulants attributed to NE are primarily due to activation of adrenergic α1 receptors (α1R). Infusion of the prototypical α1R antagonist prazosin into the PFC prevents DA release in the NAc and mice devoid of α1Rs have compromised stimulant-induced NAc DA release and are insensitive to the behavioral effects of cocaine [19,20,21]. Cocaine administration protocols that induce sensitization increase α1R levels and pharmacological antagonism with prazosin blocks the expression of acute activation and sensitization [22,23,24]. Further, our group and others have demonstrated that prazosin attenuates cocaine self-administration including procedures that model relapse to cocaine-seeking [25,26,27]. Taken together, these studies suggest that prazosin may be useful in treating cocaine dependence in humans. 

Although ample preclinical evidence supports prazosin as a possible therapy, it has a half-life of approximately 2–3 h in humans so multiple doses per day would be required to achieve a therapeutic effect thus limiting its clinical utility. A more viable alternative medication is doxazosin (DOX), an α1R antagonist with similar affinity for α1Rs as prazosin, but with a half-life of approximately 11 h making it better suited for once daily dosing [28]. More importantly, we recently confirmed the possible therapeutic utility of DOX by assessing its impact on cocaine’s subjective effects in non-treatment seeking cocaine-dependent individuals [29]. 

Preliminary clinical studies appear encouraging but the impact of DOX on the behavioral effects of cocaine in animals is presently unknown. A greater understanding of how DOX may alter cocaine’s effects in animal models is critical to further its development. Therefore, the primary goal of the present study was to assess how DOX would alter the development and expression of cocaine-induced locomotor sensitization using an established behavioral paradigm [30]. 

## 2. Results and Discussion

### 2.1. Development of Locomotor Sensitization to Cocaine (Development, Days 1–5)

**Figure 1:**
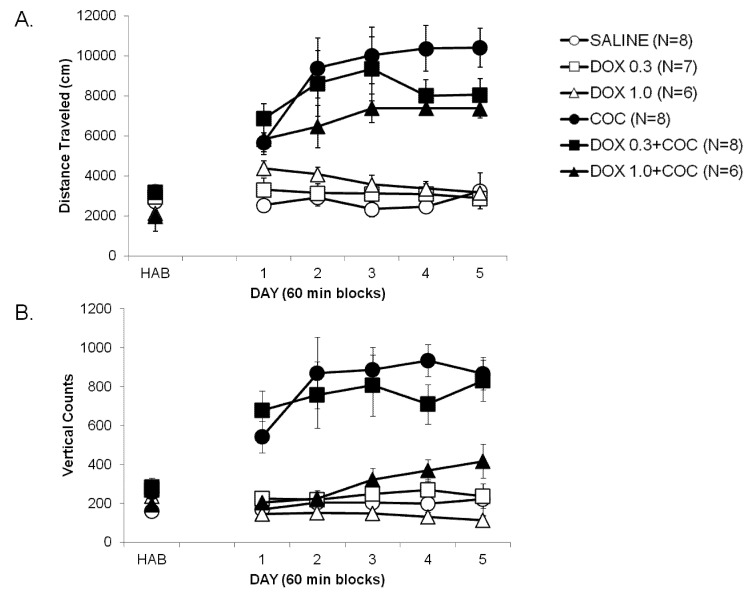
The development of cocaine locomotor sensitization is presented as mean ± standard error of mean (SEM) locomotor activity assessed as (**A**) distance traveled (cm) and (**B**) vertical counts over 5 consecutive days of drug treatments. Groups of rats (*N*) were administered vehicle (○), cocaine (●, COC; 10 mg/kg; intraperitoneally, IP), doxazosin (□, DOX; 0.3 or Δ, 1.0 mg/kg, IP), or the combination of DOX 0.3 (■) and 1.0 (▲) plus COC. DOX alone did not affect gross locomotor activity compared to saline. DOX (1.0 mg/kg) significantly blocked the development of COC-induced increases in vertical counts (**B**) across days.

Figure 1A,B shows locomotor activity and vertical counts on the habituation day (HAB) before treatments were begun and the effects of DOX on the development (Days 1–5) of cocaine-induced locomotor sensitization. Although not readily apparent from the figure, the DOX groups differed in baseline distances traveled on the HAB day before the start of the experiment. This statement is supported by a significant main effect for DOX group (*F*(2,37) = 14.04, *p* < 0.0001). Specifically, the two groups that would be administered the higher dose of DOX showed lower locomotor activity on the habituation day compared to the other groups, *p* < 0.01. There was no difference across COC groups, *p* > 0.10. Due to these baseline effects, data were also analyzed using Analysis of Co-variance (ANOCOVA). Distance traveled was greater among groups that received COC on Day 1 compared to the saline group. This is supported by a significant main effect for treatment among groups (*F*(1,37) = 34.66, *p* < 0.001). Pair-wise multiple comparisons revealed no differences among groups that received DOX alone and saline (*p*s > 0.05). Close examination of Figure 1 (Day 1) does show however, that the DOX 0.3 + COC treated group traveled greater distance than DOX 0.3 alone (*p* < 0.05).

Distance traveled among groups that received COC was greater over days compared to those that received saline. This is supported by significant main effect for COC (*F*(1,37) = 88.98, *p* < 0.0001) and Day (*F*(4,148) = 3.14, *p* = 0.05) and by the COC × Day interaction (*F*(4,148) = 5.53, *p* = 0.005). As indicated in Figure 1, repeated administration of COC increased distance traveled over days 2–5 compared to day 1 (pair-wise multiple comparisons, *p*s < 0.001) indicating cocaine-induced sensitization. Groups that received different doses of DOX + COC traveled greater distance over days compared to those that received DOX 0.3 mg/kg and 1.0 mg/kg alone or saline. This is supported by a significant interaction of COC × DOX (*F*(2,37) = 3.26, *p* < 0.05). DOX administration alone did not affect gross locomotor activity across days compared to saline; the main effect of DOX was not significant nor were any of its interaction effects, *p*s > 0.10. Results of the ANOCOVA also showed a significant main effect of COC, *F*(1,37) = 15.54; *p* < 0.0005, and a significant interaction of COC × DOX, *F*(2,37) = 7.37; *p* < 0.005. 

The number of rears (vertical counts) exhibited on the HAB day did not differ by COC or by DOX group, *p*s > 0.10. Thus, these data were not subjected to analysis by ANOCOVA. There was also no effect of COC or DOX on numbers of rears exhibited on DAY 1 (*p* > 0.01). Cocaine administration increased numbers of rears as supported by the significant COC effect, *F*(1,37) = 60.47; *p* < 0.0001. As seen in Figure 1B, the groups that were administered cocaine reared more than the groups administered saline. Numbers of rears differed across days as supported by the significant DAY effect, *F*(4,148) = 3.20; *p* < 0.5. In addition, there was a trend for the effect of COC on numbers of rears to change over days as suggested by the trend towards significance for the COC × DAY interaction, *F*(4,148) = 2.40; *p* < 0.06. In general, numbers of rears increased over days in groups administered cocaine. This is seen by comparing the numbers of rears in the COC groups on Day 1 to Day 2 (Figure 1B). Treatment with the higher DOX dose decreased numbers of rears induced by cocaine (Figure 1B). This statement is supported by the significant main effect of DOX, *F*(2,37) = 10.37; *p* < 0005, and by the COC × DOX interaction, *F*(2,37) = 5.54; *p* < 0.01.

### 2.2. Expression of Locomotor Sensitization to Cocaine (Day 15 Drug Challenge)

**Figure 2:**
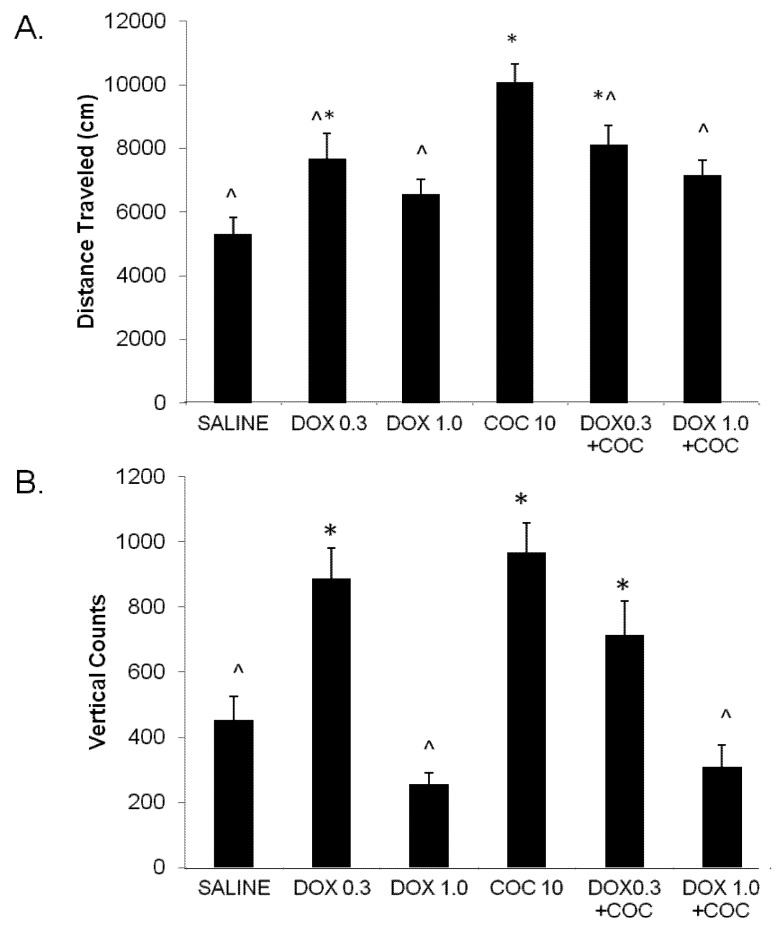
The expression of cocaine locomotor sensitization is presented as mean ± standard error of mean (SEM) locomotor activity assessed as (**A**) distance traveled (cm) and (**B**) vertical counts on the cocaine challenge test day in which all rats were administered cocaine (COC, 10 mg/kg). This test was performed 10 days after termination of the drug treatments. The groups (N) had previously received 5 consecutive days of vehicle, cocaine, doxazosin (DOX; 0.3 or 1.0 mg/kg, IP), or the combination of DOX plus cocaine. DOX significantly altered the expression of cocaine-induced sensitization as exhibited by decreased in distance traveled (**A**) and vertical counts (**B**) (DOX main effect *p* < 0.0001; COC × DOX interaction *p* < 0.001). * Significant difference from saline (*p* < 0.05) and ^ significant difference from COC 10 mg/kg (*p* < 0.05).

Ten days following cessation of drug treatments (drug washout) all rats were administered COC (10 mg/kg) to assess the expression of behavioral sensitization. As seen in Figure 2, distance traveled on day 15 differs among the treatment groups following an acute COC injection. Groups previously administered cocaine during the development phase showed greater locomotor activity than the groups that did not have this exposure. This is supported by the significant main effect of COC (*F*(1,7) = 15.54, *p* < 0.0005). This increase in expression of sensitization to the locomotor effects of cocaine was significantly reduced by DOX. This is supported by the significant COC × DOX interaction effect, *F*(2,37) = 7.37; *p* < 0.01, although the main effect of DOX failed to reach significance, *p* > 0.10.

The data on expression of sensitization in numbers of rears is shown in Figure 2B. Prior cocaine exposure tended to increase number of vertical counts (rears) as seen by the trend towards significance for the main effect of COC, *F*(1,37) = 3.47; *p* < 0.08. Prior DOX exposure significantly altered the numbers of vertical counts seen in this expression test as supported by the main effect of DOX, *F*(2,37) = 19.15; *p* < 0.0001. The COC × DOX interaction was also significant, *F*(2,37) = 9.22; *p* < 0.001. As seen in Figure 2B, prior exposure to the low dose of DOX alone led to an increase in rearing whereas prior exposure to the high dose of DOX alone led to a decrease in vertical counts relative to the saline alone group. Of the three groups with prior COC exposure, DOX had a dose-dependent effect to decrease numbers of vertical counts. This is further supported by Newman-Keuls *post hoc* analysis (distance traveled: significant difference between COC alone and DOX 0.03 + COC and DOX 1.0 + COC, *p*s < 0.05; vertical counts: significant difference between COC alone and DOX 1.0 + COC, *p* < 0.05). 

The results of the present study demonstrate that pretreatment with the high DOX dose (1.0 mg/kg) effectively blocked the development (days 1–5) and expression (day 15, challenge) of COC-induced locomotor sensitization. This effect was more robustly demonstrated for vertical counts compared to distance traveled. The ability of DOX to block sensitization to cocaine is likely due to specific antagonism of the effects of COC and not due to gross disruption of activity. This conclusion is supported by several findings in the present study. First, distance traveled and vertical counts in the DOX alone groups were no different from that of the saline group. Second, the blockade of the expression of COC-induced sensitization was dose-dependent. Finally, in groups that received DOX alone, the high dose blocked both measures of activity whereas the low DOX dose only decreased distance traveled in the expression phase suggestive of a dose-response relationship. Although we did not assess DOX blood levels, it is unlikely that attenuation of COC’s effects is due to residual drug because 10 days had elapsed since the last administration. It is more probable that DOX administration prevented long-term neuroplastic changes responsible for COC sensitization (see below). Taken together, these data are consistent with other studies similarly demonstrating that NE, and α1Rs in particular, are critical in the development and expression of COC-induced locomotor sensitization. 

Our results support data from previous studies in rodents showing that pretreatment with the prototypical α1R antagonist, prazosin, blocks the effects of COC on locomotor activation [21,22,23,24,31]. We now extend these findings to the longer-acting α1R antagonist, DOX. Numerous studies have shown attenuation of the behavioral effects of stimulants, in addition to other drugs of abuse, either by genetic negation (gene knock-outs) or pharmacological blockade of α1 receptors [21,22,23,24,31,32,33]. The ability of DOX to potently attenuate the expression of COC-induced sensitization is also consistent with Salomon *et al.* (2006) who demonstrated similar effects with prazosin on the expression of amphetamine-induced behavioral sensitization following a 1-month withdrawal period [34]. In contrast, one report found no effects of prazosin on sensitization to COC [35]. 

Prazosin administration also attenuates the ability of COC to support place conditioning [36] and induce COC-seeking in an animal model of relapse [27,36]. The discriminative stimulus effects of COC [37] are reduced and breaking points for COC self-administration decreased by prazosin [25,26,37]. Yet, two reports found no acute effects of prazosin or terazosin (a similar α1 antagonist) on COC self-administration [38,39]. Although species or procedural differences may have contributed to these divergent findings, it is more likely that chronic, not acute, α1 blockade is required to alter COC self-administration. Indeed, we found that sensitization of COC-self-administration is blocked by prazosin when administered in a similar manner as used within the present study [26]. Nevertheless, our data are still consistent with the idea that chronic α1R blockade is required to appreciably attenuate stimulant-induced effects on behavior. That is, acute DOX administration did not alter COC-induced locomotor activation (Figure 1, Day 1) whereas chronic treatment significantly attenuated the development (Days 2–5) and expression of COC sensitization (Figure 2) that was more profoundly demonstrated on vertical compared to horizontal measures (Figure 1B and Figure 2B). 

The ability of DOX to block the development and expression of COC sensitization was more robustly seen on vertical compared to horizontal activity measures. Vertical activity is generally considered a measure of stereotypy, or non-purposeful repetitive behaviors (rearing, sniffing and head-bobbing) commonly seen in rodents following stimulant administration. The mechanism(s) responsible for the specific effect of DOX on vertical behavior are unknown but may relate to the neuroanatomical distribution of α1Rs in motor circuits. Preclinical studies associate increased DA levels in the dorsal striatum/mPFC [40,41] and dis-regulated inhibitory/excitatory influence of neurons that project to the substantia nigra with COC-induced stereotypy [42,43]. α1Rs are located on pre and post-synaptic elements in the substantia nigra [44] where adrenergic agonists activate nigral neurons which is blocked by prazosin [45]. Therefore, the ability of DOX to attenuate COC-induced stereotypy measures more robustly found in the present study may relate to action at α1 receptors located in multiple brain areas (PFC and substantia nigra) that mediate this behavior. Further studies are needed to assess whether blockade of the development and expression of COC-induced stereotypy specifically, is a better indicator of possible pharmacotherapies for COC-dependence.

Disulfiram is a medication indicated for alcohol-use disorder yet numerous clinical studies have shown it modestly decreases COC use in COC-dependent individuals regardless of alcohol status [46]. We previously utilized the same behavioral protocol used in the present study to assess the effects of disulfiram on COC locomotor sensitization [30]. In contrast to DOX, disulfiram *increased* the development and expression of COC locomotor-sensitization. The mechanism responsible for the divergent effects produced by DOX and disulfiram on COC-locomotor sensitization is unknown but may be explained, in part, by dose of each drug tested and disulfiram’s complex peripheral and central pharmacological actions [47]. Regarding dose, similar to disulfiram, we found that the low dose of DOX (0.3 mg/kg) administered alone during the developmental phase appeared to pre-sensitize rats (Figure 2B). The higher DOX dose (1.0 mg/kg) however completely blocked the expression of COC-sensitization. Similar to disulfiram, DOX effects were more readily seen on vertical count measures [30]. Studies in humans also support the notion that the ability of disulfiram treatment to decrease COC’s reinforcing effects is dose dependent. Indeed, results from a laboratory-based within-subjects clinical trial indicate that lower doses of disulfiram based on a mg/kg bases increased, whereas higher doses decreased choices for cocaine [48]. These results are consistent with a recent out-patient clinical trial showing that low doses (62.5 mg/day) of disulfiram also increased, whereas higher doses (250 mg/day) slightly decreased COC use [49]. By way of copper chelation, disulfiram and its metabolite, diethyldithiocarbamate inhibit numerous peripheral and central enzymes involved in the metabolism and reinforcing effects of COC including dopamine-β hydroxylase leading to increases in DA and decreases in NE [17]. Serotonin neurotransmission and receptor levels are also increased with disufliram treatment [50,51]. Recent studies also suggest that acute disulfiram administration can augment COC-induced increases in PFC DA [52]. In contrast to the non-specific effects of disulfiram, DOX almost exclusively targets α1 receptors [28]. Taken together, it is likely dose and mechanisms of action particular to DOX and disulfiram that contribute to their divergent effects on COC sensitization. 

Enduring neuroplastic changes within mesocorticolimbic circuitry are believed to contribute to the long-lasting behavioral effects observed in COC sensitization [53,54]. COC-induced neural changes target DAergic projections from the VTA to the NAc and PFC as well as glutamatergic input acting through α-amino-3-hydroxy-5-methylisoxazole-4-propionate (AMPA) receptors from the PFC to the VTA and NAc [55,56,57,58]. All of these structures receive ample NEergic input from medullary NE cell bodies and the LC [59,60]. Recent studies have localized α1 receptors within mesocorticolimbic circuitry clarifying their complex interaction with neurotransmitters DA and glutamate known to contribute to stimulant-induced sensitization [61,62,63]. For example, activation of pre-synaptic α1Rs on PFC terminals in the VTA enhances glutamate release onto presumed DA-containing neurons and this effect is blocked by prazosin [63]. NE-induced activation of α1Rs within the PFC also increases DA, an effect that is facilitated by the adrenergic agonist phenylephrine and blocked by prazosin [64]. α1R antagonism within the NAc also attenuates COC-induced increases in DA levels and behavioral activation [61]. Taken together, the present study is congruent with numerous studies demonstrating the critical importance of NE, acting through α1Rs, in augmenting and controlling central DA neurotransmission as well as affecting neural circuits that alter the behavioral effects of stimulants in animals [19,20,21,65,66,67,68,69,70]. 

α1Rs are primarily localized on pre-synaptic elements in the VTA, NAc and PFC but some may be located post-synaptically in the NAc [44,61,62]. This neuroanatomical distribution allows NE, acting through α1Rs, to influence DA neurotransmission by way of numerous mechanisms [20,44,61,62,63]. Accordingly, dosing regimens of COC that induce sensitization potently increase α1Rs in limbic brain regions presumably to accommodate COC-induced increases in DA [70]. This suggests that long-lasting drug-induced neuroplastic changes associated with the expression of stimulant sensitization may be mediated, in part, through α1Rs. The present study is consistent with others demonstrating that α1R blockade prevents the expression of stimulant-induced sensitization even after an extended withdrawal period [34]. Taken together, evidence supports the conclusion that antagonism at α1Rs with DOX targets different brain areas to influence many pathways and neurotransmitter systems known to mediate stimulant-induced effects. Further studies investigating molecular machinery responsible for DOX’s ability to attenuate COC’s behavioral effects are needed.

## 3. Material and Methods

### 3.1. Animals

Adult male Sprague–Dawley rats (*n* = 6–8/group, Harlan Sprague-Dawley Inc., Indianapolis, IN, USA) were used in this study. Rats were housed three per cage in polypropylene cages in a temperature- and humidity-controlled room maintained on a 12:12 light/dark cycle (lights on at 7:00). Food and water were available *ad libitum*. Protocols were approved by the Baylor College of Medicine Institutional Animal Care and Use Committee and followed the “Principles of Laboratory Animal Care” (NIH publication No.85-23, revised 1996). Facilities were accredited by the American Association of Laboratory Animal Care.

### 3.2. Test Apparatus

The TruScan photobeam activity system (Coulbourn Instruments, Allentown, PA, USA) was used to measure horizontal and vertical distance traveled in centimeters. This system consisted of a clear arena (16″L × 16″W × 15.5″D) that had two sets of infrared sensors with one located at floor level and the other 2.5″ above floor level. Data from beam breaks were tabulated and analyzed by a PC computer and using a Coulbourn Instruments software system (TruScan 2.03). 

### 3.3. Drugs

Cocaine HCl (National Institute on Drug Abuse, Research Triangle Park, NC, USA) and DOX (Sigma-Aldrich, St Louis, MO, USA) were prepared in sterile saline and administered IP in a volume of 1 mg/mL by body weight (kg). Drug solutions were prepared fresh daily. 

### 3.4. Groups

To test the effects of DOX on locomotor sensitization to cocaine, six groups of rats (*n* = 6–8 per group) were employed. Groups included vehicle (saline), cocaine (10 mg/kg), DOX (0.3 and 1.0 mg/kg) and DOX + cocaine. Pretreatment time was 30 min for DOX. The dose range for DOX was chosen based on our previous studies showing that 0.3 mg/kg prazosin, a similar compound to DOX but with a lower half-life, blocked drug-induced relapse to cocaine seeking in rats [27]. A higher DOX dose was chosen based on a human clinical trial [29].

### 3.5. Locomotor Sensitization Procedure

Assessment of locomotor sensitization to cocaine was performed in a similar manner as before [30]. Rats were randomly divided into groups then administered saline, and habituated to the apparatus for 60-min daily until baseline accumulative measures did not significantly differ. The development test phase began on the next day (day 1) when rats were administered drugs according to group and immediately placed in the activity chambers for 60-min. This same procedure was followed for the next four consecutive days (days 2–5). The expression test phase was conducted 10 days later (Day 15) following a drug “washout” period when no drug administration occurred. On day 15, all rats from all six groups received cocaine injections (10 mg/kg) and activity levels were measured as before. 

### 3.6. Data Analysis

Distance traveled (cm), which reflects ambulatory activity, was the primary endpoint analyzed. Habituation locomotor activity was assessed using a two-way ANOVA (COC dose × DOX dose). Data from the development phase (days 1–5) of the locomotor study were analyzed with a 2 × 3 × 5 repeated measures ANOVA with treatment dose of cocaine (0, 10 mg/kg) and DOX dose (0, 0.3, 1.0 mg/kg) as the main factors with repeated measures over days. Because there were slight group differences in baseline activity, these measures were incorporated into an Analysis of Co-variance. Acute effects of cocaine (day 1) among the groups were analyzed separately with a 2 × 3 ANOVA representing between group factors of cocaine dose and DOX dose. Data from the expression phase (day 15) were also analyzed with a 2 × 3 ANOVA. When applicable, significant main effects were followed by *post-hoc* analysis with Newman-Keuls tests. Data are presented as mean ± SEM and significant *p* values set at <0.05.

## 4. Conclusions

We recently demonstrated that DOX blocked COC’s positive subjective effects (e.g., “high”, “stimulated”, “like cocaine” and “desire cocaine”) in non-treatment seeking, COC-dependent individuals [29]. This suggests that the basic locomotor sensitization paradigm employed in this study may have predictive validity in assessing possible pharmacotherapies for COC dependence in humans. Based on this assumption, medications that both attenuate the *development* (specifically vertical activity) and *expression* of COC-induced behavioral sensitization may be characteristics of potential pharmacotherapeutic agents for COC-dependence in humans. DOX in particular also possesses a number of beneficial pharmacological characteristics that make it a promising pharmacotherapy. For example, DOX is: (1) presently indicated for the treatment of hypertension; (2) is cardio-renal-protective; and (3) has few known drug interactions [71,72]. More importantly, DOX blocks the hypertensive and positive subjective effects of COC in humans [29]. Taken together, the present study highlights the importance of α1Rs in the behavioral effects of COC and supports further research and development of DOX as a treatment for COC-dependence in humans.
